# Assessing Differences between Clinical Isolates of *Aspergillus fumigatus* from Cases of Proven Invasive Aspergillosis and Colonizing Isolates with Respect to Phenotype (Virulence in *Tenebrio molitor* Larvae) and Genotype

**DOI:** 10.3390/pathogens11040428

**Published:** 2022-03-31

**Authors:** Sam El-Kamand, Martina Steiner, Carl Ramirez, Catriona Halliday, Sharon C.-A. Chen, Alexie Papanicolaou, Charles Oliver Morton

**Affiliations:** 1 Western Sydney University, School of Science, Campbelltown Campus, Campbelltown, NSW 2560, Australia; 17875471@student.westernsydney.edu.au (S.E.-K.); 18644337@student.westernsydney.edu.au (M.S.); 18072118@student.westernsydney.edu.au (C.R.); 2 Centre for Infectious Diseases and Microbiology Laboratory Services, Institute of Clinical Pathology and Medical Research, New South Wales Health Pathology, Westmead Hospital, Westmead, NSW 2145, Australia; catriona.halliday@health.nsw.gov.au (C.H.); sharon.chen@health.nsw.gov.au (S.C.-A.C.); 3 Marie Bashir Institute for Infectious Diseases and Biosecurity, The University of Sydney, Sydney, NSW 2145, Australia; 4 Hawkesbury Institute for the Environment, Western Sydney University, Hawkesbury Campus, NSW 2753, Australia

**Keywords:** *Aspergillus fumigatus*, virulence, pathogenesis, mealworm, *Tenebrio molitor*, aspergillosis, disease modelling

## Abstract

The fungus *Aspergillus fumigatus*, the cause of invasive aspergillosis (IA), is a serious risk to transplant patients and those with respiratory diseases. Host immune suppression is considered the most important factor for the development of IA. Less is known about the importance of fungal virulence in the development of IA including the significance of variation between isolates. In this study, isolates of *A. fumigatus* from cases diagnosed as having proven IA or colonisation (no evidence of IA) were compared in assays to measure isolate virulence. These assays included the measurement of radial growth and protease production on agar, sensitivity to UV light and oxidative stressors, and virulence in *Tenebrio molitor* (mealworm) larvae. These assays did not reveal obvious differences in virulence between the two groups of isolates; this provided the impetus to conduct genomic analysis. Whole genome sequencing and analysis did not allow grouping into coloniser or IA isolates. However, focused analysis of single nucleotide polymorphisms revealed variation in three putative genes: AFUA_5G09420 (*ccg-8*), AFUA_4G00330, and AFUA_4G00350. These are known to be responsive to azole exposure, and *ccg-8* deletion leads to azole hypersensitivity in other fungi. *A. fumigatus* virulence is challenging, but the findings of this study indicate that further research into the response to oxidative stress and azole exposure are required to understand the development of IA.

## 1. Introduction

The fungus *Aspergillus fumigatus* is a globally distributed decomposer of organic matter in the environment. It produces vast numbers of conidia, which are easily distributed by wind currents; these can be inhaled by birds and mammals, leading to the development of several disease states [1]. In humans, the most severe disease state is invasive aspergillosis (IA), which usually affects immunocompromised individuals, particularly those that are neutropenic [2,3]. Not all at-risk patients develop IA, and the severity of host-damage can vary; the disease occurs in only 7% of acute myeloid leukemia patients [4]. The contribution of variation in fungal virulence to the development of IA is poorly understood. Variation in virulence is apparent in isolates of *A. fumigatus* from lower respiratory samples of individuals who have the risk factors for IA but show no evidence of IA; these isolates are referred to as colonisers [3,5,6,7]. To date, studies have had difficulty identifying the key differences between colonising and IA isolates using in vitro tests and model hosts [5].

Intraspecies variation in *A. fumigatus* isolates has been described in traits that may be important for pathogenesis [8]. These include the growth rate [8,9], pigmentation [10], resistance to oxidative stress [11], and azole antifungals [12]. Despite these studies, inferring the clinical significance of intraspecific variation in *A. fumigatus* remains challenging. Another approach is the use of invertebrate models to study fungal infections. These models include nematodes (*Caenorhabditis elegans*), fruit flies (*Drosophila melanogaster*), and the larvae of moths (*Galleria mellonella*) and beetles (*Tenebrio molitor*) [13,14]. An advantage of *G. mellonella* and *T. molitor* larvae compared to the other invertebrate models is that they can be reared at 37 °C, the internal body temperature of humans [13]. An issue with *G. mellonella* is that it is not readily available in Australia as it is defined as a pest, making *T. molitor* an attractive alternative. The validity of these invertebrate models has been strengthened since the patterns of *A. fumigatus* virulence are consistent between invertebrate and vertebrate models [15,16]. 

Another approach to examine variation in fungal virulence is whole genome sequencing (WGS), which can be employed to identify intraspecies genetic variation in known virulence factors [17,18]. The high-resolution nature of WGS has revealed clinically relevant heterogeneity in the fungal pathogens *Candida albicans* [19] and *Cryptococcus neoformans* [20]. *C. albicans* showed major differentiating genetic variants located on genes associated with biofilm production [19], and WGS analysis of *C. neoformans* identified intra-specific heterogeneity in 40 genes putatively associated with pathogenesis [20]. 

Most studies into *A. fumigatus* intraspecific variation have focused on comparisons between environmental isolates and clinical isolates. In this study, we examined intraspecific variation in only clinical isolates of *A. fumigatus*. These came from cases defined as having no evidence of IA (coloniser isolates) and cases defined as having IA (IA isolates); cases were defined according to the European Organization for Research and Treatment of Cancer and the Mycoses Study Group (EORTC/MSG) criteria [21]. This approach was similar to a study performed using *A. fumigatus* and *A. terreus* in *Drosophila melanogaster* [5]. We adapted *T. molitor* larvae as a model system [14] to measure *A. fumigatus* virulence and used this model along with other assays (radial growth rate, resistance to oxidative stress, and infection of *T. molitor* larvae) to assess the virulence. We also used WGS to identify fungal characteristics linked to IA. This will provide a foundation for future studies into the factors that determine the outcome of the host–fungus interaction.

## 2. Results

### 2.1. Phenotypic Characterisation of Clinical A. fumigatus Isolates

**Figure 1:**
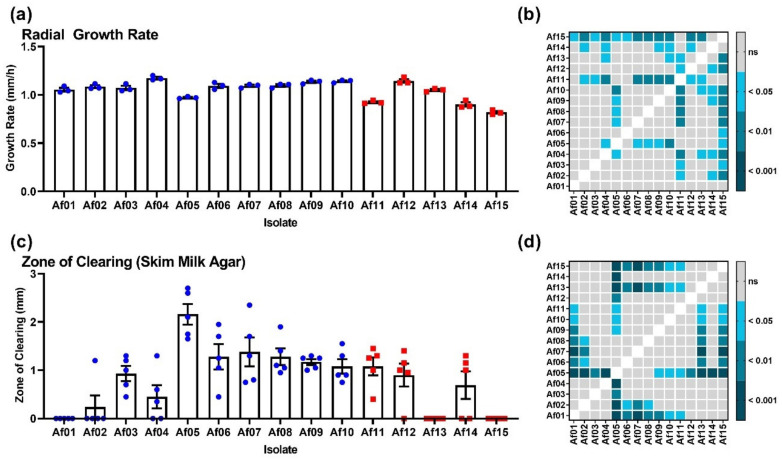
Phenotypic comparison of clinical *A. fumigatus* isolates; isolates 1–10 originated from patients with no evidence of IA, and isolates 11–15 were isolated from patients diagnosed as having IA. (**a**) Radial growth rate of coloniser and IA isolates on PDA at 37 °C; (**c**) diameter of the zone of inhibition on SMA made by colonizer and IA isolates. Blue data points are for coloniser isolates and red data points are for IA isolates. Variation amongst isolates was examined using a Welch’s ANOVA with Dunnet’s T3 post hoc analysis. Results of all vs. all post hoc testing are shown in tile plots (**b**,**d**).

**Figure 2:**
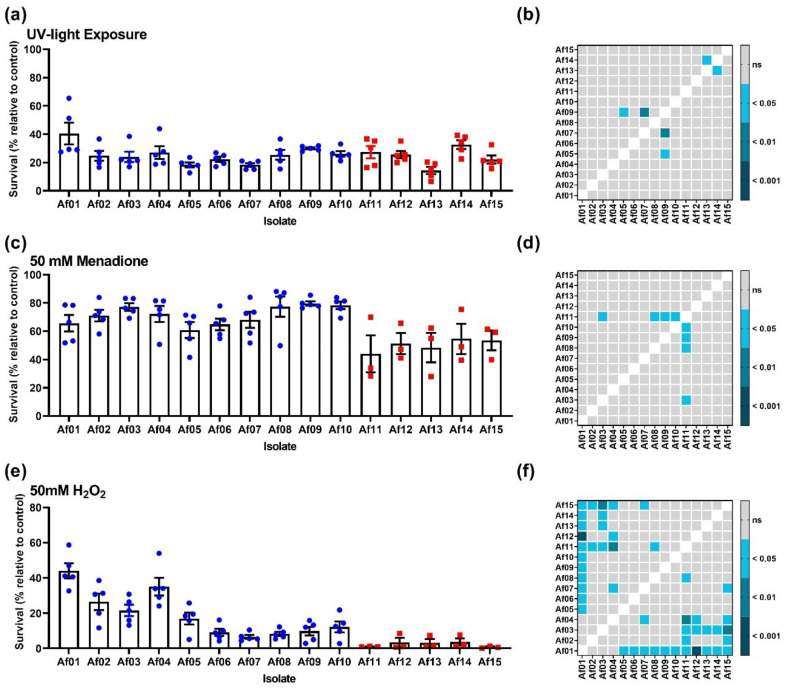
Phenotypic comparison of clinical *A. fumigatus* isolates, isolates 1–10 originated from patients with no evidence of IA and isolates 11–15 were isolated from patients diagnosed as having IA. (**a**) Survival of *A. fumigatus* conidia following 1 min of 1.6 W/m^2^ UV irradiation; (**c**) Response of *A. fumigatus* conidia to acute treatment (3 h exposure) with 50 mM menadione (**e**) Response of *A. fumigatus* conidia to acute treatment (3 h exposure) with 50 mM H_2_O_2_. Blue data points are for coloniser isolates and red data points are for IA isolates. Variation amongst isolates was examined using a Welch’s ANOVA with Dunnet’s T3 post hoc analysis. Results of all vs. all post hoc testing are shown in tile plots (**b**,**d**,**f**).

Intraspecific variation in 15 clinical isolates of *A. fumigatus* was assayed through exposure to UV light, oxidative stress, radial growth rate, and protease production (Figure 1 and Figure 2). These data indicated that IA isolates were not clearly different from coloniser isolates in the virulence-associated traits tested in this study. The coloniser isolates showed a trend towards greater fitness with respect to radial growth, UV resistance, and resistance to oxidative stress.

The radial growth rate of clinical *A. fumigatus* isolates was evaluated on potato dextrose agar (PDA) at 37 °C (Figure 1a). Growth of IA isolates displayed a trend towards slower radial growth than coloniser isolates. On average, coloniser isolates grew 124.1 (± 4.4) µm/h faster than IA isolates. The trend was consistent, with four of the five slowest growers being IA isolates (Figure 1a). There was variation in protease production between isolates (*p* < 0.0001) on skim milk agar (SMA), but there was no significant difference based on isolate origin (colonisers 997 µm ± 629 µm; IA 534 µm ± 506 µm). The production of proteases showed greater variability between isolates than radial growth rate (Figure 1c).

Coloniser and IA isolates showed similar levels of viability following one minute of UV irradiation 1.6 W/m^2^ (Figure 2a). These data suggest that conidia from IA isolates do not differ significantly from coloniser isolates with respect to UV irradiation. The response of *A. fumigatus* isolates to three hours of exposure to 50 mM H_2_O_2_ or 50 mM menadione indicated an increased resistance to oxidative stress in coloniser isolates compared to IA isolates, particularly with respect to menadione treatment (Figure 2c,e). After exposure to menadione, coloniser isolates had an average survival of 71.5% (±2.1) compared to 50.4 (±1.9) for IA isolates, whereas exposure to H_2_O_2_ led to 19% (±7.3) survival compared to 2.4% (±0.8%) for IA isolates. The data presented in Figure 1 and Figure 2 do not suggest that there are sufficient differences between colonizer and IA isolates to explain why the IA isolates were associated with cases of IA. Therefore, we also conducted virulence and genomic analyses. 

### 2.2. Modelling A. fumigatus Virulence in T. molitor Larvae

#### 2.2.1. Model Validation

**Figure 3:**
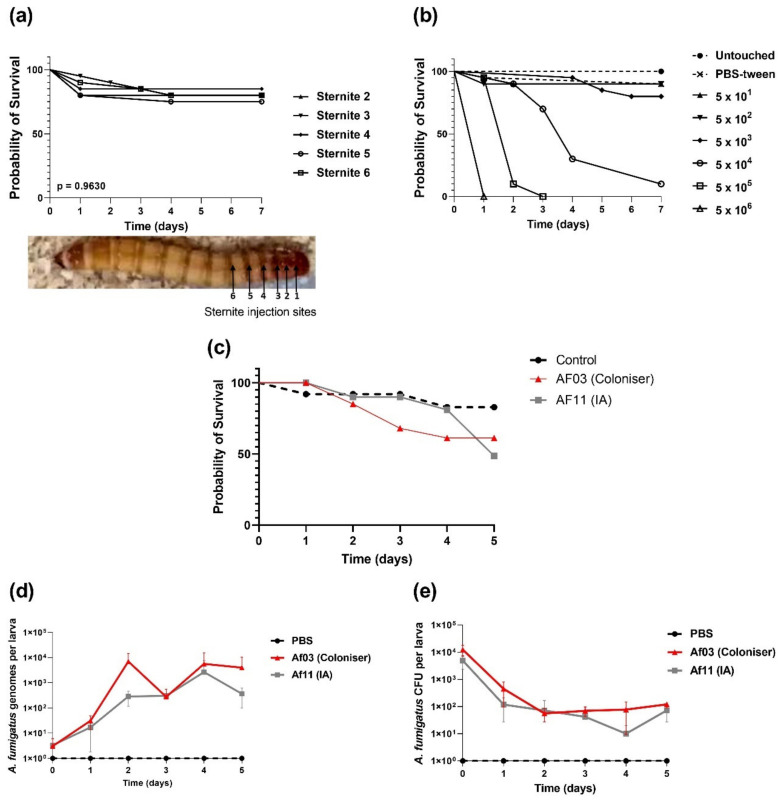
Validation of *T. molitor* larvae (mealworms) as a model of invasive fungal infections. (**a**) Survival of mealworms injected with sterile PBS-Tween (0.05% *v*/*v*) at the base of 5 different sternites; (**b**) dose-dependent survival of *T. molitor* larvae infected with *A. fumigatus* (Af01) conidia; (**c**) Kaplan–Meier survival plot of mealworms injected with 5 × 10^4^ *A. fumigatus* spores from coloniser isolate Af03, IA isolate AF11, or PBST. There were five worms at each time point for only one replicate experiment to enable optimization of fungal quantification. (**d**) Corresponding fungal load per *T. molitor* larva, expressed as *A. fumigatus* CFU per larva or (**e**) *A. fumigatus* genomes per larva measured by qPCR. Data presented for CFU and qPCR are mean and standard error for five worms.

The injection of mealworms with PBS-tween had an impact on the survival of mealworms that was similar for all injection sites (*p* = 0.96; Figure 3a). However, it was observed that injecting mealworms at the base of the fifth sternite led to the lowest frequency of hemolymph leakage, which was associated with better outcomes. The fifth sternite was chosen as the injection site for all future experiments.

Kaplan–Meier survival curves of mealworms inoculated with *A. fumigatus* (Af01) showed a dose-dependent response (Figure 3b). All inoculum sizes tested (5 × 10^1^, 5 × 10^2^, 5 × 10^3^, 5 × 10^4^, 5 × 10^5^, and 5 × 10^6^ conidia per larva) significantly decreased the survival rate relative to controls. Inoculation with 5 × 10^4^ conidia consistently yielded mortality rates above 50%, which is required for the calculation of the median survival time, without killing at a rate so high that isolates of higher virulence would be difficult to resolve. All further experiments used an inoculum of 5 × 10^4^ conidia per larva.

#### 2.2.2. Quantification of *A. fumigatus* in Infected *T. molitor* Larvae

Methods for monitoring fungal infection in the *T. molitor* larvae were evaluated (Figure 3c). Fungal quantification was attempted using both viable plate counts and quantitative PCR (qPCR). Viable plate counts (Figure 3d) showed the presence of greater amounts of the fungus, 1.3 × 10^4^ (±5.4 × 10^3^) CFU/larva earlier but lower CFU counts, 1.2 × 10^2^ (±3 × 10^1^) CFU/larva later in the experiment, suggesting clearance of the fungus despite increasing the larval mortality. qPCR analysis (Figure 2e) indicated that the fungal burden increased over time from 3.1 × 10^1^ (±2 × 10^1^) genomes/larva earlier to 4 × 10^3^ (±6 × 10^2^) genomes per larva later in the experiment, which matched the expectations of the experiment created by the mortality data (Figure 3c). The difference may be attributable to *A. fumigatus* being a filamentous fungus, leading to unreliable recovery of CFU from larvae after fungal germination and maturation into hyphae. Monitoring of infection may be resolved by microscopic analysis of the larvae, but that methodology is challenging and has not been optimized for this study.

#### 2.2.3. Quantifying Virulence of Clinical *A. fumigatus* Isolates in *T. molitor*

**Figure 4:**
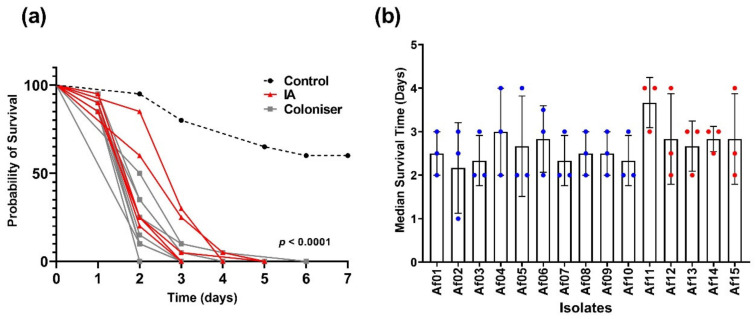
Virulence of clinical *A. fumigatus* isolates in *T. molitor* larvae. (**a**) Kaplan–Meier curve of larval infection by *A. fumigatus* (infection by both coloniser and IA isolates). The chart is a representative replicate from three replicate experiments. Control represents larvae injected with 5 µL PBS-T; the *p*-value was determined by log-rank test run on coloniser and IA isolate data. (**b**) Median survival time of *T. molitor* larvae injected with 5 × 10^4^ spores and incubated at 37 °C for 7 days. Blue data points are for coloniser isolates and red data points are for IA isolates. Data shown are mean and standard error from three replicate experiments, variation amongst isolates was examined using a Welch’s ANOVA with Dunnet’s T3 post hoc analysis (*p* = 0.6).

The isolate origin had no significant effect on the survival of infected *T. molitor* larvae (Figure 4). There was a trend for shorter mean survival times in larvae inoculated with coloniser isolates, but this was not significant. The average survival rate was 2.52 (±0.25) days with coloniser isolates and 2.97 (±0.4) days with IA isolates (Figure 3b). In this study, the shorter survival time represents greater virulence. The ability of fungi to cause death in mealworms or other invertebrates such as *Galleria mellonella* is indicative of their virulence potential in mammalian models. From these data, there is not a clear distinction in virulence between the two groups of isolates. Inclusion of patient characteristics such as antifungal treatment would be required to fully resolve the differences between coloniser and IA isolates. 

### 2.3. Genomic Analysis of Clinical A. fumigatus Isolates

#### 2.3.1. Identifying Single Nucleotide Variants (SNV)

Following the assembly of the ten sequenced genomes (Table S1), the genomes were analysed to identify SNVs unique to IA isolates. Across the six coloniser and four IA isolates, a total of 981,551 SNV sites were identified in the core-genome of *A. fumigatus* when SNVs were called against the AF293 reference genome. Filtering for bi-allelic sites in unmasked regions where the locally collinear block (LCB) length is over 200 bp resulted in a final callset of 95,999 sites. SNPeff annotation revealed a total of 19,589 putative non-synonymous mutations. 

Synonymous variants were filtered for those exclusively found in one group (coloniser or IA isolates). There were five variants present in all members of one group, which were absent in all members of the other (Table 1). These variants were all previously reported in FungiDB (release 48 beta) with an allele frequency greater than 40%, suggesting these variants are real.

The SNVs found in all IA isolates but absent in coloniser isolates were localized to three genes: AFUA_5G09420, AFUA_4G00330, and AFUA_4G00350 (Table 2). AFUA_5G09420 is annotated in FunCat as Clock controlled protein (*ccg-8*) and classified by InterProScan as Transcription factor *opi1*, a *ccg-8* homolog in yeast (Table 2). No signal peptide or transmembrane domains were predicted by SignalP5 or TMHMM. AFUA_4G00350 is not annotated in FunCat but contains a metallopeptidase domain, and protein BLAST reveals similarity to fungal sequences annotated as archaemetzincin-2. Similarly, AFUA_4G00330 is not annotated in FunCat but does have several transmembrane domains.

#### 2.3.2. Detecting Presence/Absence of Oxidative Stress Response Genes

Scanning the genome assemblies of 10 clinical *A. fumigatus* isolates for the presence or absence of nine genes involved in the melanin biosynthetic process (GO:0042438) revealed no inter-isolate variation. Presence/absence analysis of 135 oxidative stress response genes (GO:0006979) showed some variation across clinical isolates. Each isolate tested had 129–131 oxidative stress response genes present. Neither variation in the total number of genes present (Table 3) nor the occurrence patterns of individual genes (Table S2) were consistent with oxidative stress resistance.

## 3. Discussion

Many phenotypic properties of *A. fumigatus* with theoretical links to virulence show intra-specific heterogeneity [5,17,25,26,27]; however, the clinical relevance of this variation remains unclear. In this study, we tested the hypothesis that comparison of phenotypic traits related to virulence would elucidate differences between isolates that cause IA and those that just colonise patients. Fifteen clinical *A. fumigatus* were characterised with respect to the growth rate, protease production, resistance to oxidative stress, and virulence in *T. molitor* larvae. We then compared the genomes of isolates from patients that were colonised to isolates from patients that had proven IA.

The growth rate of *A. fumigatus* isolates is theorized to impact virulence as greater quantities of fungal biomass are more difficult for the immune system to clear [8,9]. In *A. fumigatus* deletion mutants, decreased virulence in murine models is often accompanied by a decreased growth rate [28,29,30]. Positive correlations between the growth rate and virulence in murine models have also been observed in populations of wild-type isolates [9,26,31]. These findings span growth rates calculated using solid and liquid cultures, and both minimal and nutrient rich media. The growth rate does not always positively correlate with virulence, as has been demonstrated by studies in both insect and murine models [32]. As in previous studies [8], the isolates examined here showed significant variability in the growth rate, as assayed on PDA (Figure 1a,b). There was no clear difference between coloniser and IA isolates, which was also observed for the growth and creation of zones of clearing on SM agar (Figure 1c,d).

Resistance of *A. fumigatus* conidia to solar UV radiation and UV-induced reactive oxygen species (ROS) is important for the survival of airborne conidia. However, there was very limited intraspecific variation in UV resistance observed in this study (Figure 2a,b). The limited significant variation in UV resistance is in coloniser isolates and therefore would not be considered essential for the development of IA [8,11]. Conidia do contain molecules associated with fungal pathogenesis such as melanin, which may be important for the establishment of infection by *A. fumigatus* [33]. Conidial cell wall melanin has been implicated in pathogenesis by promoting pre-germination concealment of immunogenic PAMPs [34,35], evasion of internalization by phagocytes [36], and persistence within immune and alveolar epithelial cells [37,38]. Further studies that focus on the role of melanin in infection would be required to determine the role of melanin in the differential development of IA. Analysis of the response to oxidative stress revealed greater resistance in coloniser isolates than in IA isolates (Figure 2c–f). A link between resistance to oxidative stress and virulence has been demonstrated previously in *A. fumigatus*; deletion of catalases and superoxide dismutases has been associated with reduced or delayed virulence in murine models, and sensitization to killing by phagocytes [39]. It seems counter-intuitive for our isolates to demonstrate the inverse association. However, this is similar to the overall trend in our observations of growth rate (Figure 1) and virulence in *T. molitor* larvae (Figure 4). 

Hosts susceptible to IA are almost always immunodeficient; however, their immunological profiles can vary wildly [40]. IA is both uncommon and often misdiagnosed, making it difficult to obtain a large set of clinical isolates standardised with respect to potentially noise-creating host factors such as the primary condition, therapeutic history, or geographical region. The use of animal models (mouse and invertebrate) allows the virulence of *A. fumigatus* isolates from different hosts to be compared in an experimental system where host factors are standardized. In this study, we validated and optimized *T. molitor* larvae (mealworms) as a model for invasive fungal infection. Unlike *Drosophila* or nematodes, mealworms can be reared at 37 °C. They can also be inoculated via injection, allowing for precise control over the infective load. Advantages over *G. mellonella* include low maintenance and widespread availability, mitigating the requirement of maintaining in-house colonies. These advantages of *T. molitor* have also been reviewed elsewhere [41]. Here, we have shown that the injection of mealworms with *A. fumigatus* isolates causes mortality in a dose-dependent manner (Figure 3b). Furthermore, the fungus is present in the mealworm throughout the course of experimentation (Figure 3d,e). The mean survival time for *G. mellonella* injected with 10^5^ conidia of wild-type *A. fumigatus* was 2–3 days [16], which compares well with the data from this experiment (Figure 4). Although our inoculum was 10^4^ conidia, differences could be expected due to the isolates used and the immunity systems of the respective hosts.

Several studies comparing clinical and environmental *A. fumigatus* isolates have been conducted. In immunosuppressed mice, environmental isolates were found to be less virulent than clinical isolates using mortality-based metrics [42]. A similar trend was observed in mixed infection murine models, where mice were co-infected with a clinical and corresponding environmental isolate and relative virulence inferred from the ratio of recovery after the mice shows signs of pulmonary distress [43]. Importantly, this trend in virulence is not mammal specific. Clinical isolates are also more virulent than environmental isolates in *G. mellonella* larvae [25]. Taken together, these studies suggest that virulence data produced in animal models are clinically relevant and that either (1) some environmental *A. fumigatus* isolates possess phenotypic profiles more conducive to causing infection than others, or that (2) within a human host, virulence-enhancing micro-evolution occurs.

In *T. molitor* larvae, IA isolates could not be distinguished from coloniser isolates (Figure 4a,b). This suggests that clinically important fungal properties selected for use in human hosts were not selected for our invertebrate model. One possibility is that virulence factors important in overcoming a clinical barrier to infection have fitness costs that become visible when the selective pressure is lifted due to differences between the clinical environment and the experimental system used to assess virulence. The ability of an isolate to survive prophylaxis or response therapy is clinically important; this was not modelled in *T. molitor* larvae. Significant fitness trade-offs associated with resistance to common antifungals, including those used in prophylactic and first-line treatment of IA, have been observed in *Candida* [44,45]. Notably, some of the common azole-resistance conferring mutations do not appear detrimental to *A. fumigatus* fitness in either immunosuppressed [46] or immunocompetent mice [47].

To guide future work, we explored the genomic variation in clinical isolates and shortlisted variants likely to be of interest (Table 1). Previously, broad-scale phylogenetic comparison and even geneset-restricted SNV-based analysis has failed to resolve different clinical forms of aspergillosis [45,46]. In this study, we identified five non-synonymous SNVs that were found in all IA isolates but not in coloniser isolates. The SNVs are all common in FungiDB [48], suggesting that they are legitimate genetic variants and not sequencing artifacts. Identical allele frequencies for four of these variants implies that they represent a haplotype common in clinical isolates. The shortlisted variants affected three genes: AFUA_5G09420, AFUA_4G00330, and AFUA_4G00350. 

These were further investigated through a literature search to determine their functions (Table 2). AFUA_5G09420 is transcription factor CCG-8. Knockout studies in *Neurospora crassa* and *Fusarium verticillioides* showed that loss of CCG-8 hypersensitizes cells to azole antifungals, and this can be rescued by transformation with a CCG-8 containing vector [22]. AFUA_4G00350 and AFUA_4G00330 are less well characterized. AFUA_4G00350 is likely to be a metallopeptidase (a possible archaemetzincin-2 homolog), as classified by InterProScan. AFUA_4G00330 contains several transmembrane domains and likely encodes a membrane-bound protein (Table 2). There is some evidence that itraconazole treatment leads to increased expression of both AFUA_4G00330 and AFUA_4G00350 [23,24].

The potential relationship between AFUA_5G09420, AFUA_4G00330, and AFUA_4G00350 with responses to azole fungicides led us to test the isolates against a range of fungicides. This analysis indicated potential differences in response to some fungicides by IA isolates (Figure S1). This will require further investigation since further replication and testing will be required to confirm a role for differential drug responses in IA isolates. The MIC methodology will also require optimization as differences in drug sensitivity will be subtle since the tested isolates did not appear to have obvious azole resistance alleles. Strong drug resistance may not be required to enable the development of IA since consistent drug concentrations are not always observed between or within patients [49,50]. There will be patients or regions of the body with sub-optimal drug concentrations that will allow the growth of fungal variants with low/intermediate drug resistance.

In addition to SNV analysis, we examined the presence/absence of genes involved in oxidative stress response or the melanin biosynthetic process in the genomes of clinical *A. fumigatus* isolates. The patterns of variation observed were not consistent with patterns of oxidative stress resistance (Table 3; Table S2). Thus, it is unlikely that gene presence/absence drives oxidative stress resistance. Previous studies have also failed to resolve *A. fumigatus* isolates of differing clinical significance based on the presence/absence of virulence-associated genes (including those involved in oxidative stress response) [51]. There is evidence that *Saccharomyces cerevisiae* has distinct responses to oxidative stress induced by menadione and H_2_O_2_ [52], and this has been supported by transcriptional analysis of *Aspergillus oryzae* [53]. In the *A. oryzae* study, similar oxidative stressors led to different transcriptional responses in the important transcription factors *yap1* and *skn7*, as well as catalases and superoxide dismutase. The *A. fumigatus yap1* is important for the oxidative stress response by controlling the expression of downstream genes. Understanding differences in oxidative stress response between fungal isolates will require a functional genomics approach that incorporates genomic, proteomic, and transcriptomic data. 

Testing coloniser and IA isolates of *A. fumigatus* with assays for virulence was inconclusive, but genome comparison indicated that understanding the development of IA in at-risk patients will require further study into complete or partial azole resistance.

## 4. Materials and Methods

### 4.1. Isolates of A. fumigatus and Media

All strains of *A. fumigatus* were provided by the Centre for Infectious Diseases and Microbiology Laboratory Services, Institute of Clinical Pathology and Medical Research, Westmead Hospital (Table 4). Each case was classified according to the criteria of the European Organization for Research and Treatment of Cancer and the Mycoses Study Group (EORTC/MSG) [21]. Fifteen isolates of *A. fumigatus* were studied; ten isolates (Af1–Af10) were colonisers, from patients with no evidence of IA [3,5,6,7], and five (Af11–Af15) were invasive isolates, from patients with proven IA [5,21]. Cultures were grown on PDA for three days at 37 °C and conidia suspensions were prepared from each isolate as previously described [54]. Conidial concentrations were determined using a Neubauer chamber and viability was determined using CFU counts of ten-fold conidial dilutions on PDA (Sigma-Aldrich, Castle Hill, Australia) plates that were incubated for 24 h at 37 °C [55]. New conidial suspensions were prepared for each replicate experiment and concentrations were tested before the experiments.

### 4.2. Phenotypic Variation Amongst Clinical A. fumigatus Isolates

#### 4.2.1. Radial Growth Rate and Proteolysis on SMA

The radial growth and growth rate have been associated with fungal virulence in several studies, with faster growth being indicative of greater virulence [26,56,57]. For each *A. fumigatus* isolate, a PDA plate (90 mm diameter) was spot-inoculated with 10^4^ conidia. Cultures were incubated at 37 °C for 3 days. The colony diameter was measured at regular intervals. The radial growth rate was calculated by plotting the colony diameter (mm) versus time (h) from five linearly distributed data points; the growth rate was the slope of this line. Three independent experiments were performed.

Proteolytic activity has been associated with infection and tissue invasion in pathogenic fungi [58]. A simple method to determine the proteolytic activity is to grow microbes on a medium such as SMA where the proteolysis of milk proteins creates a zone of clearing around the colony [59,60]. SMA was prepared by adding 250 mL UHT skim milk to 250 mL autoclaved, still molten, 4% agar (Sigma-Aldrich). The milk and agar were mixed and poured into Petri dishes. These were inoculated with 10^4^ conidia to the centre of the Petri dish. Cultures were incubated at 37 °C for 4 days. The zone of inhibition was measured daily to determine the time where the greatest discrimination between isolates was observed; a wider zone of clearing indicates greater proteolytic activity. Three independent experiments were performed.

#### 4.2.2. Conidial UV Resistance

Conidial melanin has an important role in protection against UV light and the initial interactions between host and *A. fumigatus*, the conidial melanin providing protection against the activity of host phagocytes [61,62]. In this study, we used the response to UV to determine whether there were isolates with defects in conidial melanin. For each isolate, approximately 200 conidia were plated onto malt extract agar (MEA) (Sigma-Aldrich). Five plates inoculated with the same isolate were placed at different positions within a TopSafe PC2 Biosafety (Bio-Air, Pero, Italy) cabinet and UV irradiated (1.6 W/m^2^) for 1 min. This was repeated for all 15 isolates. Following irradiation of each isolate, the biosafety cabinet was vented for 5 min to prevent reactive oxygen species (ROS) accumulation. Colony forming units (CFUs) on control plates were counted following incubation at 37 °C for 24 h. An additional incubation for 24 h at 25 °C preceded CFU counting of UV-irradiated plates. The percent survival for each isolate was calculated relative to a non-irradiated control and based on the average CFU counts across the five irradiated plates. The experiment was repeated four times, with the irradiated isolates being changed to achieve a uniform average UV-order position amongst all isolates.

#### 4.2.3. Measurement of Response to Oxidative Stress

To measure the effects of acute exposure to oxidative stress, isolates of *A. fumigatus* were exposed to H_2_O_2_ and menadione (both from Sigma-Aldrich) for three hours [63,64]. H_2_O_2_ (30% solution) and menadione (10 mM stock in ethanol) were added to *A. fumigatus* spore suspensions from each isolate; 1 × 10^6^ conidia/mL was incubated for 3 h at 37 °C with 0 mM, 50 mM H_2_O_2_ or 50 mM of menadione. Acute exposure required greater exposure doses than used in other studies. After incubation, the conidial viability was determined using dilution plate counts on PDA (Sigma-Aldrich); plates were incubated at 37 °C for 24 h and counted to determine CFU/mL. The percent survival was calculated relative to the 0 mM control. 

### 4.3. Using T. molitor to Measure A. fumigatus Virulence

The use of *T. molitor* larvae was based on a study using these larvae to monitor the virulence of *C. albicans* and *C. neoformans* [14]. Mealworms were purchased from BioSupplies (Biosupplies, Yagoona, Australia) and checked for uniform size (100–150 mg) and colour (dark brown to black indicates ill health) before use. In all experiments, mortality was determined by response to physical stimulation. Where not explicitly specified, mealworms were incubated in Petri dishes (rearing density: 10 mealworms/58 cm^2^ Petri dish) with 3 mL rearing diet and a slice of frozen carrot for moisture (0.4 cm^3^; 500 mg; changed daily). Rearing diet comprised of wheat bran and LSA (linseeds, sunflower seeds, and almonds) in a 5:1 *v*/*v* ratio.

#### 4.3.1. Optimisation and Validation of *T. molitor* Larvae as Models of Fungal Infection

The site of injection into *T. molitor* larvae was optimized to reduce mortality due to physical trauma. Mealworms were injected between sternites with a maximum 5 µL of liquid using a Hamilton syringe (701 N, 10 μL capacity) [14]. Groups of 20 mealworms were injected ventrally with 5 μL of PBST at the base of one of five sternites. Sternites 2–6 were tested (Figure 1a). Survival was checked daily over 7 days of incubation at 37 °C. Each treatment group included 20 mealworms. Based on the findings of this experiment, mealworms were injected at the base of sternite 5 in all virulence assays. 

To identify the fungal load with the greatest potential for resolving inter-isolate variation, a dose–response experiment was run. Groups of 20 mealworms were inoculated with 0, 5, 5 × 10^1^, 5 × 10^2^, 5 × 10^3^, 5 × 10^4^, 5 × 10^5^, or 5 × 10^6^ conidia of isolate AF01. Mortality was monitored daily over 7 days of incubation at 37 °C. The experiment was performed twice.

Groups of 30 mealworms were inoculated with 5 µL PBST (vehicle control), or 5 × 10^4^ spores from coloniser AF03 or invasive isolate AF11. Each day, 5 mealworms were sampled from each cohort for fungal load quantification. 

#### 4.3.2. Quantification of *A. fumigatus* Infection of *T. molitor* Larvae

Both infected and uninfected larvae were frozen and stored at −20 °C after virulence had been measured. Frozen larvae were decapitated and cut below the 8th sternite to provide a section of worm that would be halved (longitudinally). One half was used for viable plate counts and the other was used for qPCR.

For viable plate counts the larval section was homogenized in PBS (Sigma-Aldrich) with a disposable pestle in a 1.5 mL microcentrifuge tube. The contents were serially diluted and 100 µL was spread using an L-shaped spreader on Petri dishes containing potato dextrose agar containing chloramphenicol (Himedia, Mumbai, India). Plates were prepared in duplicate and incubated at 37 °C until colonies were visible, approximately 20 h.

For qPCR DNA was isolated from the other half of the larval section. DNA was isolated using the ISOLATE II Genomic DNA Kit (Bioline, Eveleigh, Australia) with the following modifications: larval sections were placed in a 1.5 mL microcentrifuge tube with lysis buffer (GL), this was homogenized with a micro pestle. Proteinas K was added and the homogenate incubated at 55 °C for 2 h. Glass beads (710–1180 µm and 425–600 µm) were added to 250 µL in a 2 mL screw cap tube. The homogenate was added to the 2 mL tube and the sample was disrupted in a Mini-Beadbeater (BioSpec Products, Bartlesville, USA) for two cycles of 1 min of beadbeating and 1 min on ice. The manufacturer’s instructions were then followed with an elution volume of 50 µL.

qPCR was performed on a 7500-FAST real-time PCR system (ThermoFisher Scientific, North Ryde, Australia) using primers targeting the ITS region of *A. fumigatus* as previously described [65]. The number of fungal genomes was determined compared to a standard curve of genomic DNA isolated from 10^5^ conidia.

#### 4.3.3. Measuring Inter-Isolate Variation in Virulence of *A. fumigatus*

The virulence of all 15 clinical *A. fumigatus* isolates was evaluated. For each isolate, 20 mealworms were inoculated with 5 × 10^4^ conidia (in 5 µL PBST) at the base of sternite 5. Larvae were incubated for 7 days at 37 °C. Each experimental replicate included three control groups: (1) mealworms injected with sterile PBST, (2) mealworms pierced at sternite 5 but with no solution injected, and (3) mealworms chilled on ice but otherwise untreated. 

### 4.4. Genomic Variation Amongst Clinical A. fumigatus Isolates

#### 4.4.1. DNA Isolation

For each of 10 clinical isolates, malt extract broth (20 mL) was inoculated with 10^4^ conidia and incubated for 4–5 days at 37 °C with shaking. Fungal biomass was isolated through vacuum filtration and stored at −20°C until use. Genomic DNA was extracted from biomass using the ISOLATE II Genomic DNA Kit (Bioline) with pre-lysis steps supplemented by mechanical disruption and RNA degradation. Lysis buffer (180 µL) and 100 mg of biomass was added to FastPrep Lysing Matrix G (MP Biomedicals, Seven Hills, Australia) before bead milling in a FastPrep-24 (MP Biomedicals) (max speed; 30 s). RNase A (1 µL of a 20 mg/mL) solution was then added, and samples incubated at 37 °C for 30 min. RNase was degraded by adding 25 µL of Proteinase K solution and incubating at 56 °C for 1 h. Secondary lysis steps and column clean-up were conducted as per the standard protocol. 

#### 4.4.2. Library Preparation and Sequencing

WGS library preparation (TruSeq DNA PCR-Free, Opentrons, New York, NY, USA) and sequencing (Illumina NovaSeq 6000) was conducted by the Ramaciotti Center for Genomics.

#### 4.4.3. Genome Assembly

FastQC1 (v0.11.2) was used to evaluate the read quality. The leading 7 bp, final base, Illumina adapters, and low-quality leading and trailing bases (phred < 30) were removed using trimmomatic2 (v0.38, http://www.usadellab.org/cms/index.php?page=trimmomatic (accessed on 1 June 2020)). Reads were error-corrected with LIGHTER3 (v1.1.2) [66]. SPAdes4 (v3.13) [67], was used to produce de novo assemblies of the 10 *A. fumigatus* isolates. SPAdes contigs were scaffolded into chromosomal level assemblies with Ragout6 (v2.2) [68]. Ragout scaffolding was based on synteny with the public AF293 reference genome (GCF_000002655.1), the draft genome of A1163 (GCA_000150145), and the contig-level isolate assemblies of the clinical isolates examined in this study. SPAdes contigs unplaced by Ragout were run through a nucleotide BLASTN (v2.6.0) similarity search [69]. Hits were filtered and sorted by e-value and percent identity (E < 1 × 10^−10^, >90% identity). Unplaced contigs longer than 200 bp with BLASTN top hits against *A. fumigatus* were incorporated into the assembly. The completeness of assemblies was evaluated using BUSCO, and contiguity using the n50stats.pl script packaged with the Just Annotate My genome^5^ (JAMg) pipeline (Apr 2016). Contiguity stats were generated assuming a true genome size of 29.4 Mb.

#### 4.4.4. Variant Analysis

parSNP10 (v1.2) [70], was used to align the assembled *A. fumigatus* genomes AF293 reference and call SNPs in genomic regions that align across all genomes and the reference. Harvest tools (v1.2) [70], was used to convert data to VCF format. Variants were filtered using VCFtools (v0.1.15) [71], to include only bi-allelic sites and thin mutations less than 10 bp away from the nearest SNP. SNPs were annotated based on the RefSeq annotation of the AF293 reference genome using SnpEff (v 4.3t) [72].

#### 4.4.5. Investigating Biological Function of Mutated Genes

To investigate the biological function of selected genes, associated FunCat annotations were identified using FungiFun (v2.2) [73]. Protein sequences were run through a protein BLAST. InterProScan (v5) [74], was used to classify proteins into families and predict protein domains, including signal peptide sequences using SignalP (v5) [75] and transmembrane domains using mobius and TMHMM.

#### 4.4.6. Detecting Presence/Absence of Oxidative Stress Response Genes

To investigate whether gene presence/absence explains observed differences in the oxidative stress resistance of clinical *A. fumigatus* isolates, each genome assembly was scanned for the presence of genes involved in either melanin biosynthesis (GO:0042438) or oxidative stress response (GO:0006979). The nucleotide sequences of each gene in the AF293 reference genome were extracted using the GO search functionality of EuPathDB (release 49 beta) [76]. Gene presence/absence in each assembly was evaluated using ABRicate (v1.0.1, https://github.com/tseemann/ABRicate, (accessed on 10 march 2020)) [77]. Genes were considered present if both coverage and percent identity was greater than 90%.

### 4.5. MIC Determinations

Minimum inhibitory concentrations (MICs) of several antifungal drugs were determined using Yeast Sensititre YO10 plates (ThermoFisher Scientific) as per the manufacturer’s instructions [78]. In brief, fungal conidia were added to the included broth as described in the manufacturer’s instructions to achieve a density of 1.5–8 × 10^3^ cells/mL; 100 µL of broth containing conidia was added to each well of the YO10 Sensititre plate (ThermoFisher Scientific). After inoculation, the plates were incubated at 37 °C for 24–48 h. The YO10 Sensititre plate contains the following drugs (concentration range): Amphotericin B (0.12–8 µg/mL), Anidulafungin (0.015–8 µg/mL), Caspofungin (0.008–8 µg/mL), Fluconazole (0.12–256 µg/mL), 5-Flucytosine (0.06–64 µg/mL), Itraconazole (0.015–16 µg/mL), Micafungin (0.008–8 µg/mL), Posazonazole (0.008–8 µg/mL), and Voriconazole (0.008–8 µg/mL).

## Figures and Tables

**Table 1:** SNVs that occur in IA isolates but not in Coloniser.

Chromosome	Position ^1^	Mutation ^2^	Gene	Transcript	Class	AA Change	AF ^3^
NC_007198.1	2422543	T>C	AFUA_5G09420	rna-XM_748604.1	missense	p.Thr502Ala	0.49
NC_007197.1	89009	C>T	AFUA_4G00330	rna-XM_741330.1	missense	p.Gly11Glu	0.41
NC_007197.1	95331	G>A	AFUA_4G00350	rna-XM_741328.1	missense	p.His142Tyr	0.41
NC_007197.1	95364	C>G	AFUA_4G00350	rna-XM_741328.1	missense	p.Glu131Gln	0.41
NC_007197.1	95399	G>A	AFUA_4G00350	rna-XM_741328.1	missense	p.Ala119Val	0.41

^1^ 1-based; ^2^ AF293 allele > alternative allele; ^3^ frequency of alternate allele in FungiDB.

**Table 2:** Investigating the function of genes potentially important for development of IA.

Property	AFUA_5G09420	AFUA_4G00350	AFUA_4G00330
FunCat Protein	Clock controlled protein (CCG-8)	None	None
FunCat Category	Cell type differentiation	None	None
InterProScan Protein family	Transcription factor OPI1	Peptidase M54, archaemetzincin-2. Metallopeptidase domain	None predicted.
Literature	Knockouts in *N. crassa* and *Fusarium verticillioides* hypersensitise to azoles [22]	Increased expression following itraconazole treatment [23,24].	Increased expression following itraconazole treatment [23,24].
Phobius/TMHMM	None	None	3 TMhelix, 4 Phobius transmembrane domains predicted

**Table 3:** Presence/absence of genes involved in oxidative stress response (GO:0006979) and melanin biosynthesis (GO:0042438) in the genomes of 10 clinical *A. fumigatus* isolates.

Clinical Origin	Isolate	Number of Genes Present in Assembly	
		Oxidative Stress Response	Melanin Biosynthesis
Coloniser	Af01	130	9
	Af02	130	9
	Af03	130	9
	Af04	131	9
	Af06	131	9
	Af10	129	9
Proven IA	Af11	131	9
	Af12	130	9
	Af13	131	9
	Af14	130	9

**Table 4:** Isolates of *A. fumigatus* used in the study sourced from Centre for Infectious Diseases and Microbiology Laboratory Services, Institute of Clinical Pathology and Medical Research, Westmead Hospital.

Isolate Name	Patient Classification	Isolate Origin
Af01	Coloniser ^1^	Sputum
Af02	Coloniser	Sputum
Af03	Coloniser	BAL ^3^
Af04	Coloniser	BAL
Af05	Coloniser	Sputum
Af06	Coloniser	Tissue ^4^
Af07	Coloniser	Tissue
Af08	Coloniser	Sputum
Af09	Coloniser	Sputum
Af10	Coloniser	Sputum
Af11	Proven IA ^2^	Tissue
Af12	Proven IA	BAL
Af13	Proven IA	Tissue
Af14	Proven IA	BAL
Af15	Proven IA	Tissue

^1^ Coloniser cases had no evidence of IA [3,6,7]. ^2^ Patients classified according to the EORTC/MSG criteria [21]. ^3^ Bronchoalveolar lavage. ^4^ Cultured from a tissue biopsy.

## Data Availability

The majority of the data are contained within the article or Supplementary Materials. The genome data may be available on request but will require permission from Westmead Hospital.

## Supplementary Materials

The following supporting information can be downloaded at: https://www.mdpi.com/article/10.3390/pathogens11040428/s1, Figure S1. MIC values determined for each of the coloniser (10 isolates) and IA (5 isolates) *A. fumigatus* isolates used in this study. The figure shows the drugs where there were differences in mean MIC between coloniser and IA isolates based on the Sensititre YO10 results. The horizontal lines represent the mean value of the isolates in each group; one Sensitrite YO10 plate was performed for each isolate. Table S1. Assembly Statistics for 10 *A. fumigatus* isolates and the public AF293 reference genome (GCF_000002655.1; ASM265v1). Table S2 Presence/absence of oxidative stress response genes (GO:0006979) in clinical *A. fumigatus* isolates.
