# Supplementary material for: Assessing Differences between Clinical Isolates of Aspergillus fumigatus from Cases of Proven Invasive Aspergillosis and Colonizing Isolates with Respect to Phenotype (Virulence in Tenebrio molitor Larvae) and Genotype
Source: Pathogens. 2022 Mar 31;11(4):428. doi: 10.3390/pathogens11040428 (PMC9029132; doi:10.3390/pathogens11040428)
Supplement: Supplementary file 1 [file pathogens-11-00428-s001.zip › pathogens-1643418-supplementary/Table S1.pdf]

Table S1. Assembly Statistics for 10 *A. fumigatus* isolates and the public AF293 reference genome (GCF_000002655.1; ASM265v1).

Isolate	NG_{50}	NG_{50} length	Assembly Size (bp)	Complete and single-copy BUSCOs (%)
Af01	4	3,980,904	28,694,393	98.3
Af02	4	4,042,954	28,863,661	98.2
Af03	4	4,020,486	29,139,223	98.3
Af04	4	3,908,787	28,743,407	98.3
Af06	4	3,898,399	28,754,711	98.4
Af10	4	4,043,438	29,129,594	98.3
Af11	4	3,927,423	28,724,611	98.3
Af12	4	4,000,533	29,056,945	98.3
Af13	4	4,001,372	28,916,749	98.4
Af14	4	3,881,678	29,158,005	98.3
Af293	4	3,948,441	29,384,958	98.3
