# Supplementary material for: Assessing Differences between Clinical Isolates of Aspergillus fumigatus from Cases of Proven Invasive Aspergillosis and Colonizing Isolates with Respect to Phenotype (Virulence in Tenebrio molitor Larvae) and Genotype
Source: Pathogens. 2022 Mar 31;11(4):428. doi: 10.3390/pathogens11040428 (PMC9029132; doi:10.3390/pathogens11040428)
Supplement: Supplementary file 1 [file pathogens-11-00428-s001.zip › pathogens-1643418-supplementary/Table S2.pdf]

Table S2. Presence / absence of oxidative stress response genes (GO:0006979) in clinical *Aspergillus fumigatus* isolates.

**Clinical Origin**	**Isolate**	**Proportion of gene covered (%)**[1]		
		Afu1g08790	Afu4g00180	Afu5g04050
Colonisation	Af01	99.74	100	absent
	Af02	100	100	absent
	Af03	99.9	absent	100
	Af04	99.71	100	100
	Af06	99.69	100	100
	Af10	absent	100	absent
**IA**	**Af11**	100	100	100
	**Af12**	99.69	100	absent
	**Af13**	100	100	100
	**Af14**	100	100	absent

[1] All genes in AF293 implicated in oxidative stress response (GO:0006979) that also have a variable occurrence pattern across the 10 clinical *A. fumigatus* isolates are shown.
