# Supplementary figures and images for: Assessing Differences between Clinical Isolates of Aspergillus fumigatus from Cases of Proven Invasive Aspergillosis and Colonizing Isolates with Respect to Phenotype (Virulence in Tenebrio molitor Larvae) and Genotype
Source: Pathogens. 2022 Mar 31;11(4):428. doi: 10.3390/pathogens11040428 (PMC9029132; doi:10.3390/pathogens11040428)

**Amphotericin B**

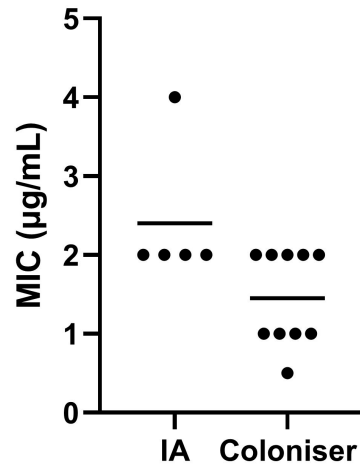

**Anidulafungin**

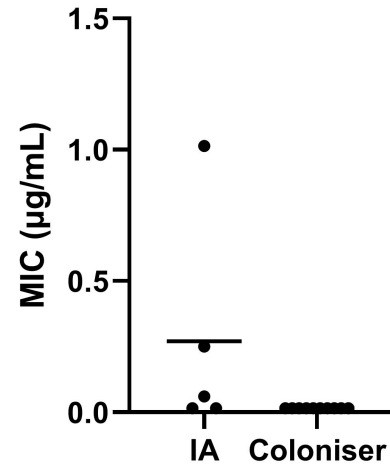

**Micafungin**

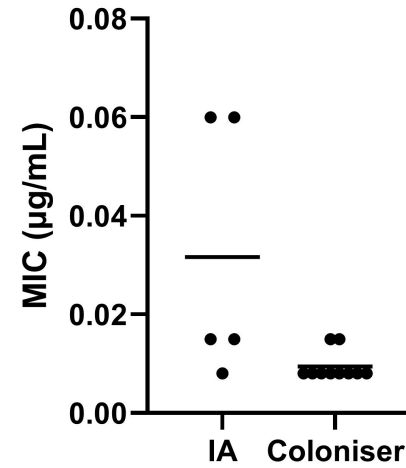

**Voriconazole**

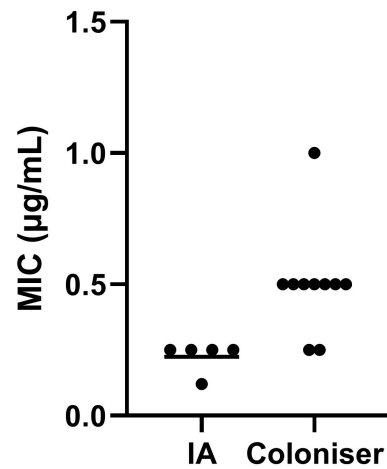

**Itraconazole**

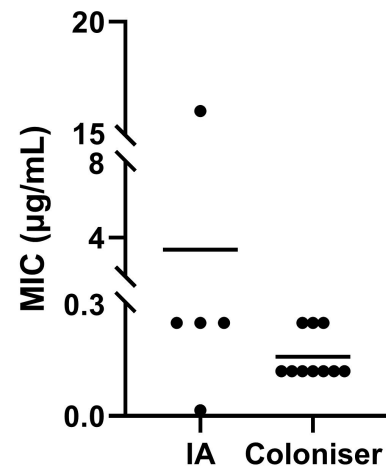

**Posaconazole**

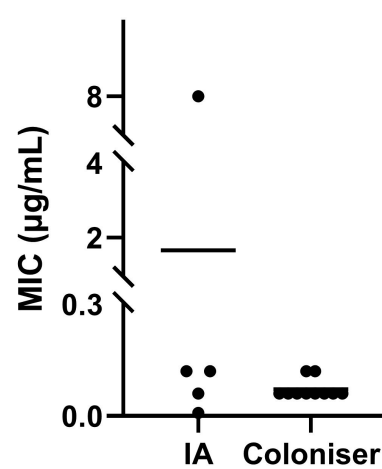

Supplement: Supplementary file 1 [file pathogens-11-00428-s001.zip › pathogens-1643418-supplementary/Figure S1.pdf]
